# The complete chloroplast genome sequence of *Cathetus clarkei* Hook.f.1890 R.W.Bouman, 2022 (Phyllanthaceae)

**DOI:** 10.1080/23802359.2025.2456184

**Published:** 2025-01-20

**Authors:** Qirong Liu, Shuang Wang, Min Fan

**Affiliations:** College of Pharmacy, Dali University, Dali, China

**Keywords:** *Cathetus clarkei*, chloroplast genome, phylogenetic tree

## Abstract

*Cathetus clarkei*, a significant folk medicinal plant, is utilized to treat a variety of ailments. In this study, we reported the complete chloroplast genome sequence of this species. The length of the complete chloroplast genome was 155,810 bp, included a pair of inverted repeat (IR) regions (26,340 bp), a large single-copy region (LSC, 84,853 bp), and a small single-copy region (SSC, 18,277 bp). It comprised 128 genes, including 83 protein-coding genes, 37 tRNA genes, and eight rRNA genes. The total GC content was 36.8%. The phylogenetic tree revealed that *C. clarkei* had a close relationship with *P. cochinchinensis*, followed by *P. reticulatus*. This study provides a reference for important medicinal plants within the Phyllanthaceae, and we can gain a deeper understanding of how the species adapts to changes in the environment, helping us to identify and protect it.

## 1. Introduction

The species of the family Phyllanthaceae are predominantly tropical in distribution and include herbs, shrubs, and trees (Hoffmann et al. 2006). This family comprises two subfamilies (Phyllanthoideae and Antidesmatoideae), 10 tribes, 57 genera, and 2050 species (Christenhusz and Byng 2016). *Phyllanthus*, a vast genus within the Phyllanthaceae family, consisting of more than 700 species, can be classified into 11 subgenuses (Mao et al. 2016). The plants of the genus *Phyllanthus* have long been used in folk medicine to treat kidney and urinary bladder disturbances, intestinal infections, diabetes, and hepatitis (Calixto et al. 1998). *Cathetus clarkei*, as a traditional medicinal plant, boasts a spectrum of pharmacological benefits, with its most notable being its potent antiviral and antibacterial capabilities. Originally categorized within the *Phyllanthus* genus, has been reassigned to its own monophyletic genus (*Cathetus*), reflecting a more accurate representation of its evolutionary lineage (Bouman et al. 2022), and its complete chloroplast genome has not been published, which leads to its systematic genetic location being unclear. Therefore, in this study, we assembled the complete chloroplast genome to improve the molecular investigation of genetic diversity and phylogenetic relationships.

## 2. Materials and methods

**Figure 1.**
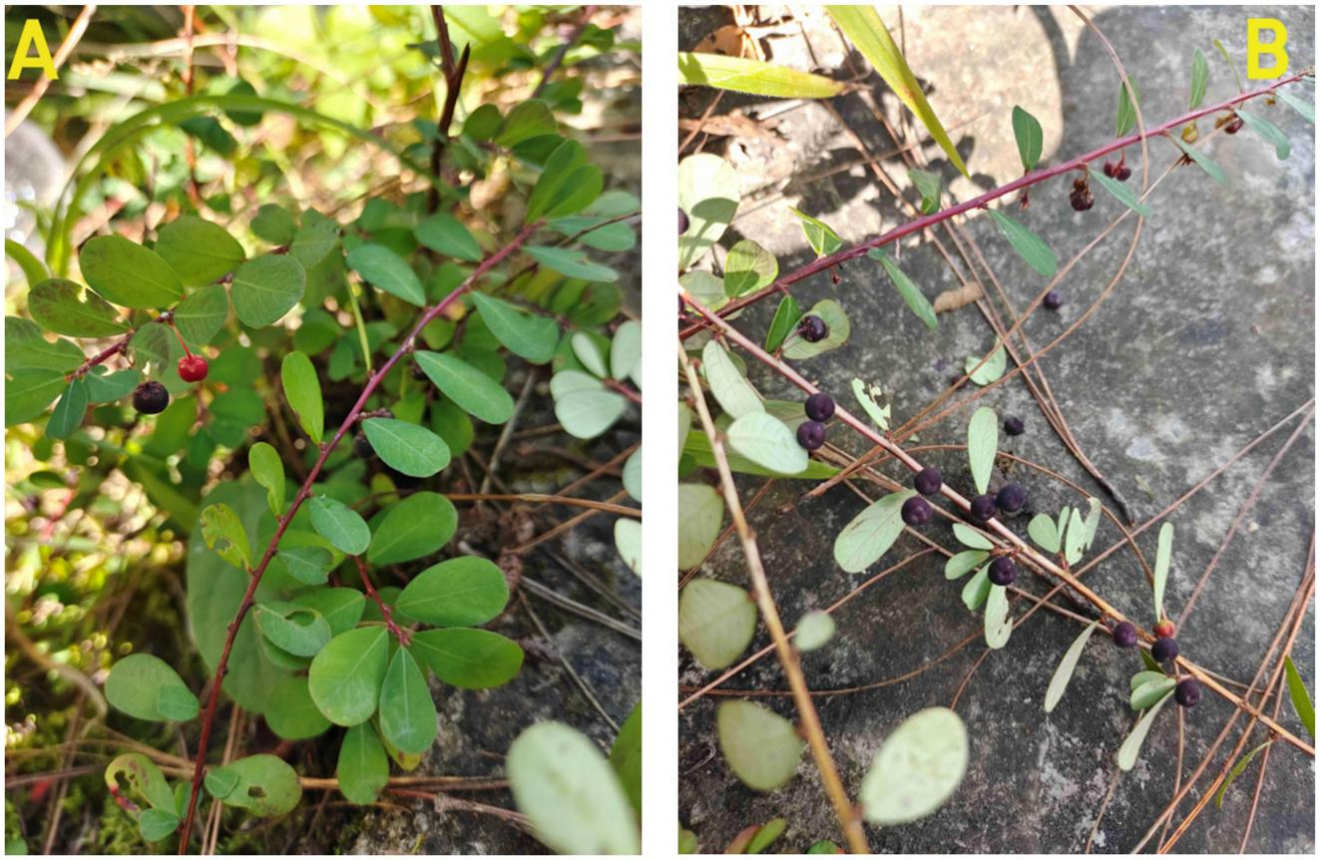
Reference images of *C. clarkei* were taken by Min Fan at Xizhou town, Dali city, Yunnan province, China. It grows mainly in Mountain forests at altitudes of 800-3000 m or in sandy shrubland near Rivers. (A) Plant panorama, and (B) the fruit of *C. clarkei.*

We collected fresh and healthy leaves of *C. clarkei* (Figure 1) in November 2022 from Xizhou town, Dali City, Yunnan Province, China (geospatial coordinates 23°85′03″N, 100°13′06″E). Min Fan both identified original plants and shot images. The specimen was deposited in the Herbarium of Dali University (http://yxy.dali.edu.cn/yhxy/, Min Fan, fanmin302301@163.com) under the voucher number FM2022111811. The total genomic DNA was extracted using the cetyltrimethylammonium bromide (CTAB) method (Doyle 1987). Sequencing was performed using the Illumina NovaSeq 6000 platform (Modi et al. 2021). At the time of sequencing, the DNA concentration was measured at 4.66 (ng/µL), with a total quality of 0.16 (µg).

The raw data consists of 5.67 GB. These reads were de novo assembled by NOVOplasty (Nicolas et al. 2017), annotated by Geneious Prime v2021.2.2 (Kearse et al. 2012), and the chloroplast genome map using CPGview (Liu et al. 2023).

To estimate the phylogenetic position of *C. clarkei*, 20 complete chloroplast genome sequences were downloaded from the National Center for Biotechnology Information (NCBI), including 18 Phyllanthaceae and two outgroup plants (*Ixonanthes chinensis* and *Hevea brasiliensis*). The common genes of 21 chloroplast genomes were aligned by MAFFT v7 (Katoh and Standley 2013), using IQ-TREE to construct a maximum-likelihood phylogenetic tree (Minh et al. 2020) and bootstrap was performed 1000 replicates. The best-fitting model was determined by ModelFinder (Kalyaanamoorthy et al. 2017) and was TVM+F + R3. Numbers at the nodes represent bootstrap support values (%).

## 3. Results

**Figure 2.**
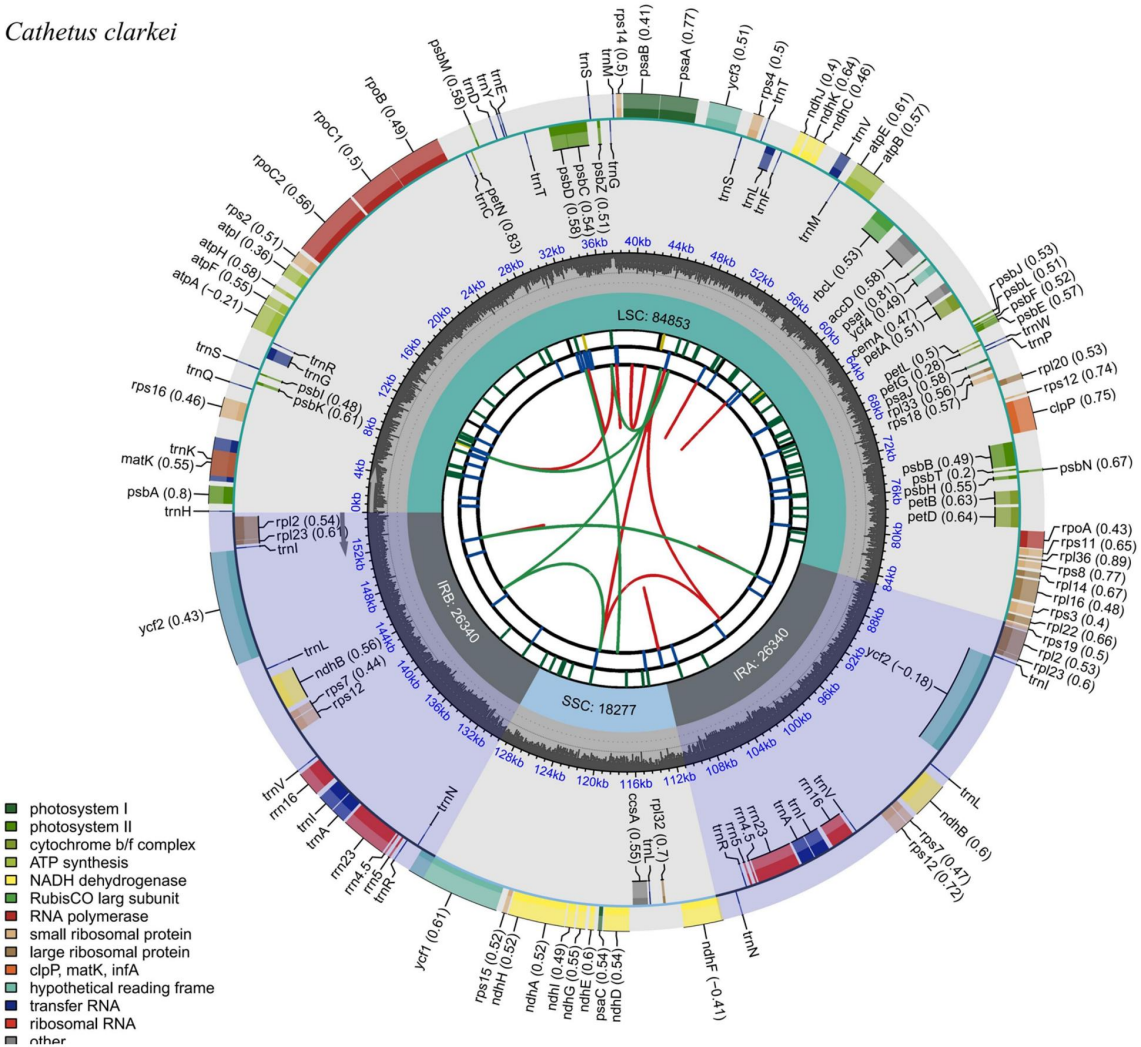
The chloroplast genome map of *Cathetus clarkei*. Genes inside and outside the circle are transcribed clockwise and counter-clockwise. The different colored boxes in the outermost circle show the genes. The inner circle has a grey area indicating the GC content, while the quadripartite structure (LSC, SSC, IRA, and IRB) is shown on the inner circle accordingly.

**Figure 3.**
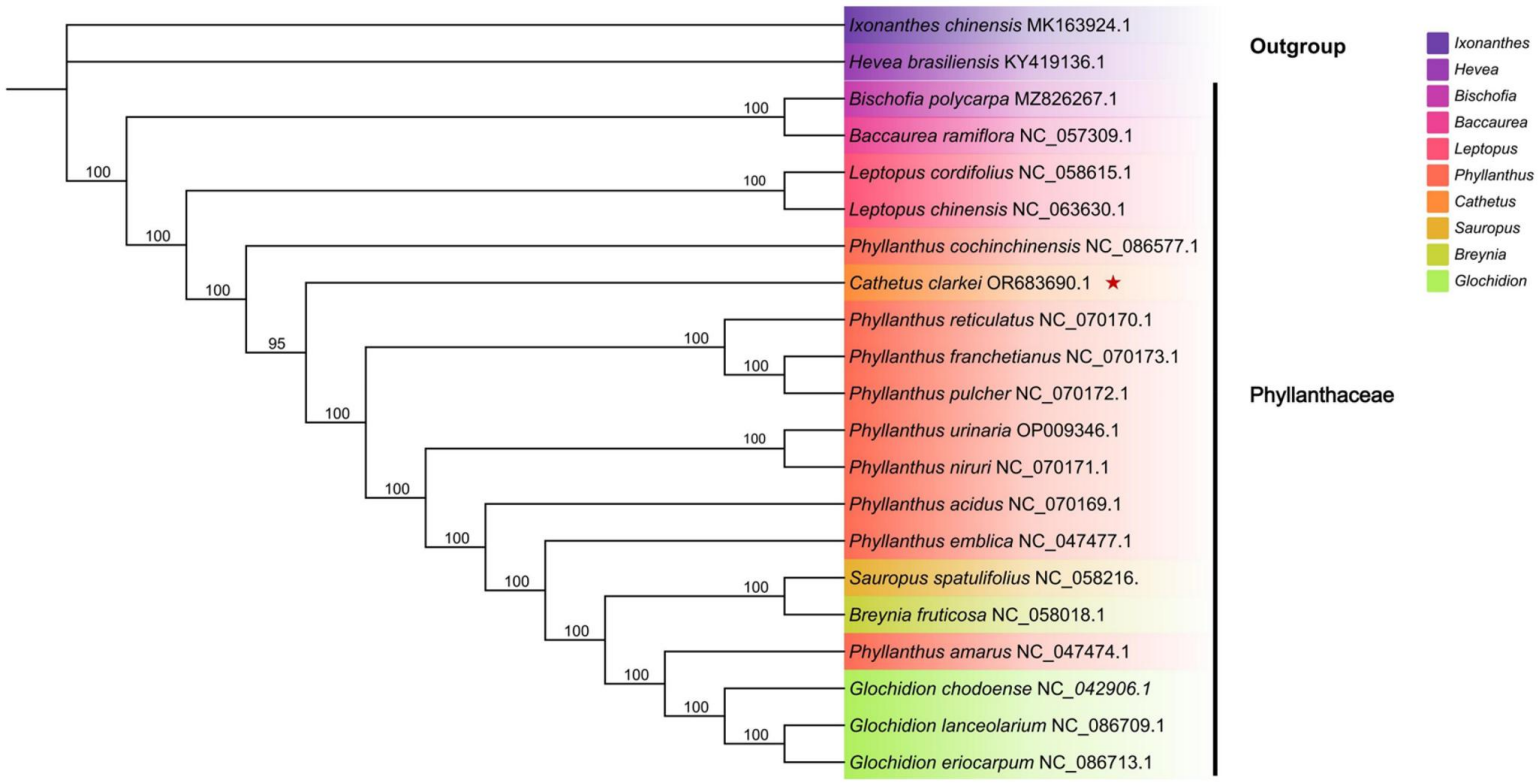
Maximum likelihood phylogenetic tree based on 21 complete chloroplast genome sequences from related species. The following sequences were used: *Ixonanthes chinensis* MK163924.1 (Zhi et al. 2019), *Hevea brasiliensis* KY419136.1 (Tangphatsornruang et al. 2011), *bischofia polycarpa* MZ826267.1, *Baccaurea ramiflora* NC_057309.1 (Hu et al. 2021), *L. cordifolius* NC_058615.1 (Rehman et al. 2021), *leptopus chinensis* NC_063630.1 (Zhao and Yu 2022), *P. cochinchinensis* NC_086577.1 (Zhang et al. 2022), *C. clarkei* OR683690.1, *P. reticulatus* NC_070170.1, *P. franchetianus* NC_070173.1, *P. pulcher* NC_070172.1, *P. urinaria* OP009346.1, *P. niruri* NC_070171.1, *P. acidus* NC_070169.1 (Danh et al. 2023), *P. emblica* NC_047477.1 (Mahajan et al. 2023), *S. spatulifolius* NC_058216.1 (Cai et al. 2020), *B. fruticosa* NC_058018.1 (Zhou et al. 2020), *P. amarus* NC_047474.1, *G. chodoense* NC_042906.1 (Cheon et al. 2019), *G. lanceolarium* NC_086709.1, *G. eriocarpum* NC_086713.

The Mean base depth map indicated a highly reliable genome assembly, with an average depth of 5083.32 and extreme depths of 8502× and 555×, respectively (Supplementary Figure 1). The length of the complete chloroplast genome of *C. clarkei* was 155,810 bp. It presented a typical quadripartite circular structure (Yang et al. 2020), which included a pair of inverted repeat (IR) regions (26,340 bp), a large single-copy region (LSC, 84,853 bp), and a small single-copy region (SSC, 18,277 bp). Besides, the complete chloroplast genome consisted of 83 protein-coding genes, 37 tRNA genes, eight rRNA genes (*rrn*16*, rrn*23*, rrn*4.5*, rrn*5S*, rrn*5S*, rrn*4.5*, rrn*23*, rrn*16), and for a total of 128 genes. The total GC content of the complete genome was 36.8% (Figure 2). Among the protein-coding genes, eight protein-coding genes have one intron (*rps*16, *rpo*C1, *pet*B, *pet*D, *rpl*16, *rpl*2 (×2), *ndh*B (×2), *ndh*A), and two protein-coding genes contained two introns (*ycf*3 *and clp*P), like other plants of this genus, the *atp*F gene does not have introns (Supplementary Figure 2). Additionally, we identified the trans-splicing gene (*rps*12), the architecture of which is depicted in (Supplementary Figure 3). The phylogenetic analysis revealed that *C. clarkei* was taxonomically classified within the Phyllanthaceae, and had a close relationship with *P. cochinchinensis*, followed by *P. reticulatus.* In the realm of rigorous statistical principles, a bootstrap value exceeding 95 is deemed necessary for reliability. In our phylogenetic tree, each node boasts a bootstrap value surpassing 95, indicating robust support for the branches (Figure 3).

## 4. Discussion and conclusions

This study has established a molecular framework that elucidates the phylogenetic relationships within the Phyllanthaceae family, contributing valuable genetic insights to the advancement of systematic research in this group. Our phylogenetic analysis has delineated distinct relationships among the Phyllanthaceae, highlighting that *C. clarkei* shared a closer evolutionary relationship with the *Phyllanthus* species. In the recently revised phylogenetic classification of the tribe Phyllantheae, *Cathetus* and *Macraea* were identified as sister branches, which contrasts with our phylogenetic tree (Bouman et al. 2022). This discrepancy arises because the revised classification integrates both genetic data and morphological characteristics, whereas our study solely relies on genetic data, lacking morphological studies. The chloroplast genome structure and gene repertoire of *C. clarkei* exhibit a striking similarity to those reported in previous studies, with a single circular DNA composed of two copies of an inverted repeat that separates the LSC region and SSC region. Notably, the absence of introns in the *atp*F gene in *C. clarkei* aligns with the majority of species within the family, except *Baccaurea ramiflora*, which occupies a basal position and retains this intron (Rehman et al. 2021), underscoring the conserved nature of the chloroplast genome across the Phyllanthaceae. This stable genetic structure favors the survival of the species capable of flexibly adapting to environmental changes. Chloroplast genome sequences have unveiled significant genetic and structural diversity among and within plant species, offering valuable insights into their evolutionary relationships (Henry et al. 2016). This valuable information is essential for a deeper understanding of how plants with medicinal properties can adapt to climate change. That helps us identify and protect these species. The taxonomic classification within Phyllanthaceae remains a subject of debate, with *Phyllanthus* identified as a polyphyletic group (Kathriarachchi et al. 2005), indicating the need for ongoing taxonomic revision and clarification.

## Supplementary Material

Supplementary.docx

## Data Availability

The data that support the finding of this study are openly available in GenBank of NCBI at https://www.ncbi.nlm.nih.gov, reference number OR683690.1 for *C. clarkei*. The associated BioProject, BioSample, and SRA numbers are PRJNA1156236, SAMN43486704, and SRR30535823, respectively.
