# Supplementary material for: The complete chloroplast genome sequence of Cathetus clarkei Hook.f.1890 R.W.Bouman, 2022 (Phyllanthaceae)
Source: Mitochondrial DNA B Resour. 2025 Jan 20;10(2):130–3. doi: 10.1080/23802359.2025.2456184 (PMC11749125; doi:10.1080/23802359.2025.2456184)
Supplement: Supplementary.docx [file TMDN_A_2456184_SM1701.docx]

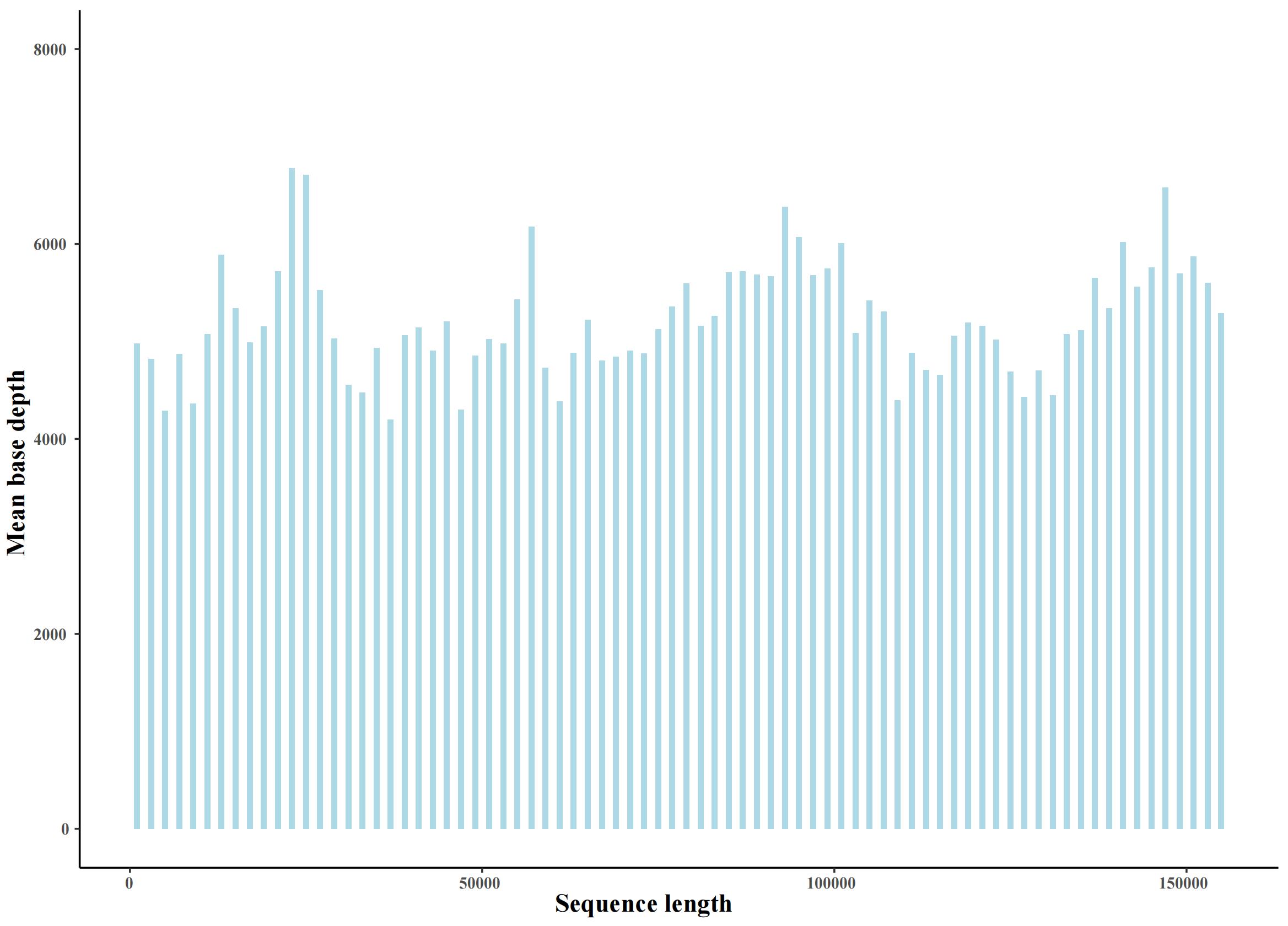
Supplementary Figure 1 Mean base depth of the chloroplast genome assembly of *Cathetus clarkei*.

The average depth=5083.3×

The maximum depth=8502×

The minimum depth=555×


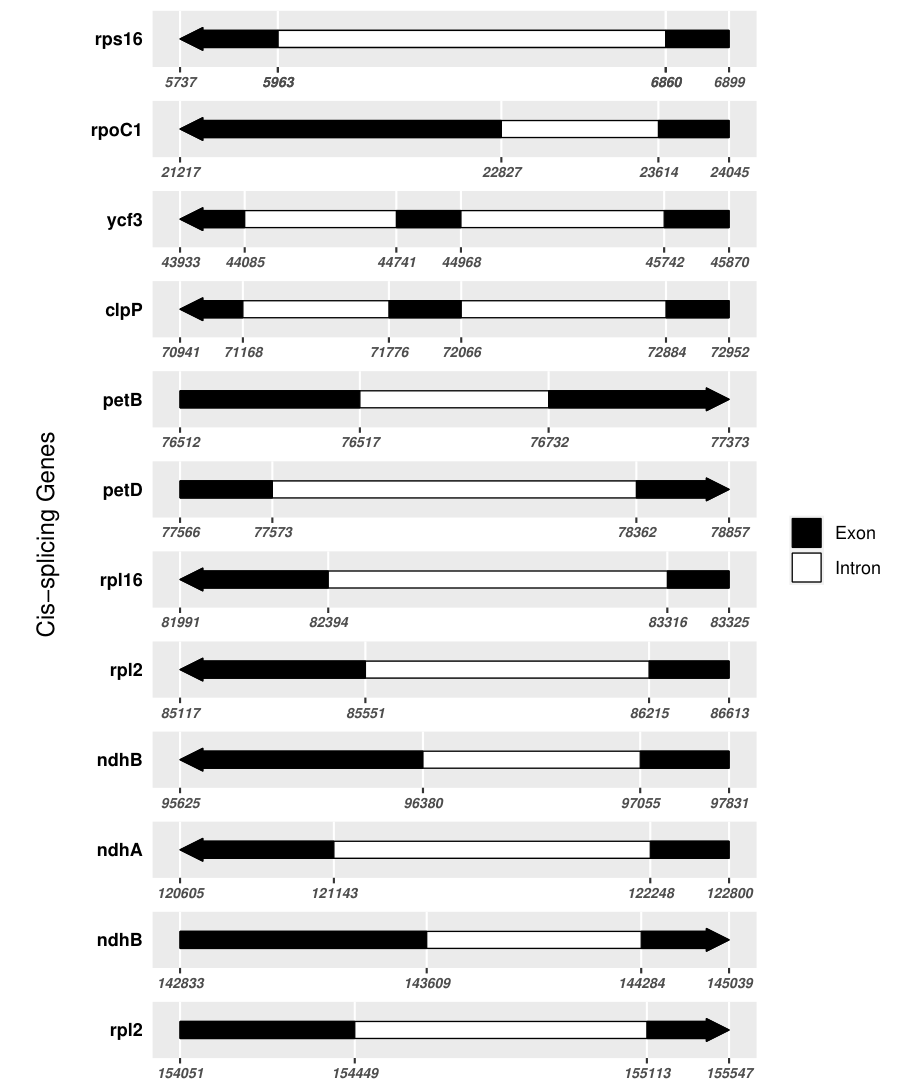


Supplementary Figure 2 Schematic map of the cis-splicing genes in the *Cathetus* *clarkei* chloroplast genome.


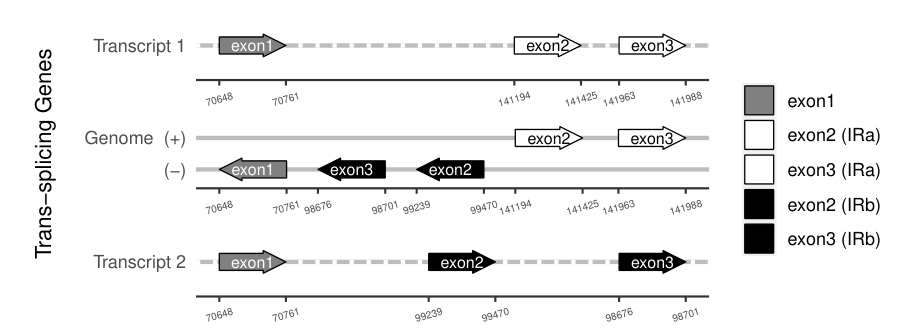


Supplementary Figure 3 Schematic map of the trans-splicing gene *rps*12 in the *Cathetus clarkei* chloroplast genome.
